# Supplementary material for: Evolving stem cell fate capacities and transcriptional priming in the developing olfactory epithelium
Source: bioRxiv. 2026 Jul 29:2026.07.28.741343. Preprint. [Version 2] doi: 10.64898/2026.07.28.741343 (PMC13484052; doi:10.64898/2026.07.28.741343)
Supplement: Supplementary file 1 [file NIHPP2026.07.28.741343v2-supplement-1.pdf]

bioRxiv preprint doi: https://doi.org/10.64898/2026.07.28.741343; this version posted July 29, 2026. The copyright holder for this preprint (which was not certified by peer review) is the author/funder. This article is a US Government work. It is not subject to copyright under 17 USC 105 and is also made available for use under a CC0 license.

Supplemental Figures

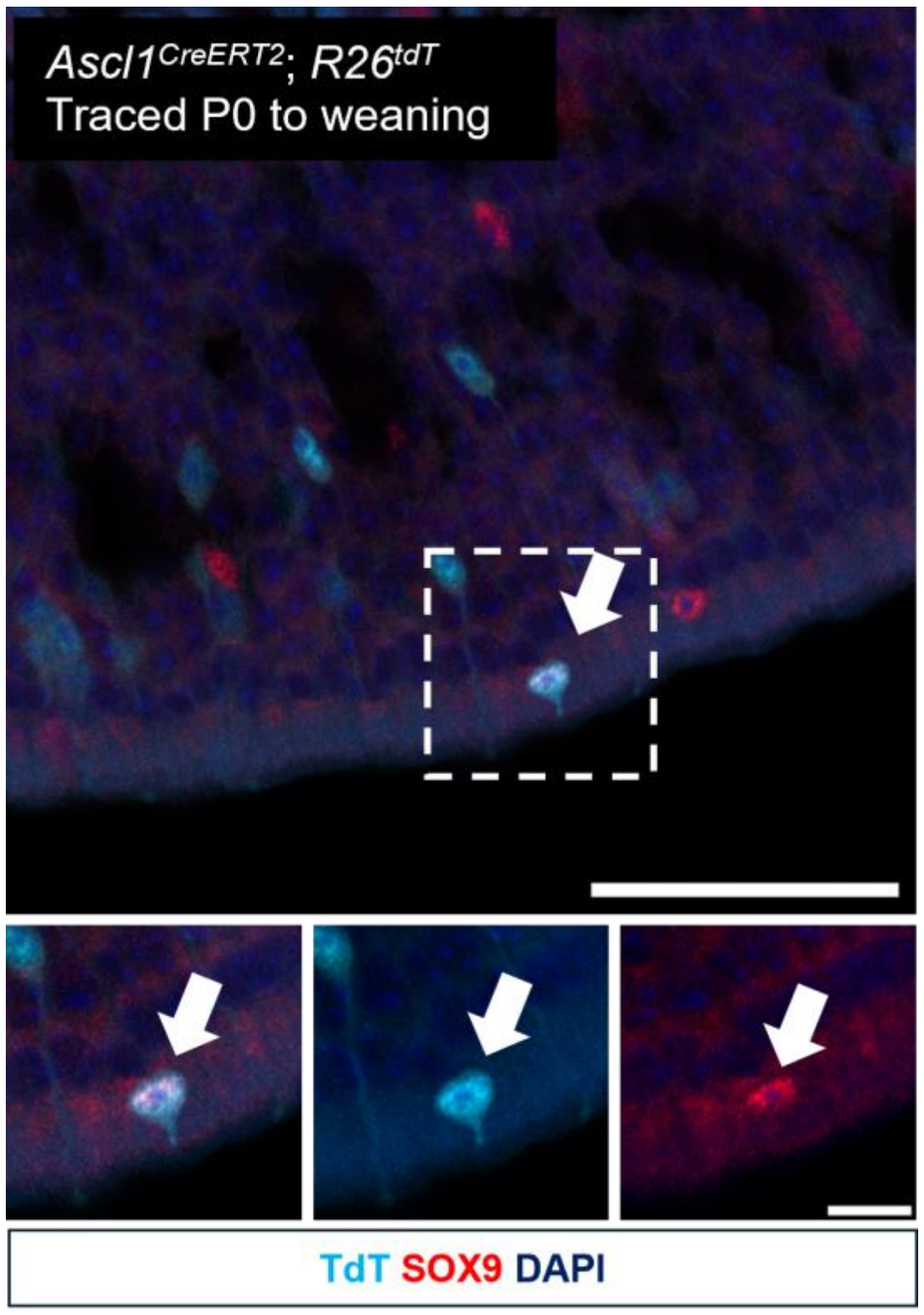

Figure S1: Microvillus cell labeled by *Ascl1*$^{CreERT2+}$ lineage tracing. An example of a rare tdT^{+} (cyan) microvillus cell (SOX9^{+}; magenta) traced from P0 and visualized at weaning. Arrow and boxed region indicate the traced cell; lower panels show higher-magnification views of the boxed region. Scale bar, top panel: 50 µm; inset panels: 10 µm.

bioRxiv preprint doi: https://doi.org/10.64898/2026.07.28.741343; this version posted July 29, 2026. The copyright holder for this preprint (which was not certified by peer review) is the author/funder. This article is a US Government work. It is not subject to copyright under 17 USC 105 and is also made available for use under a CC0 license.

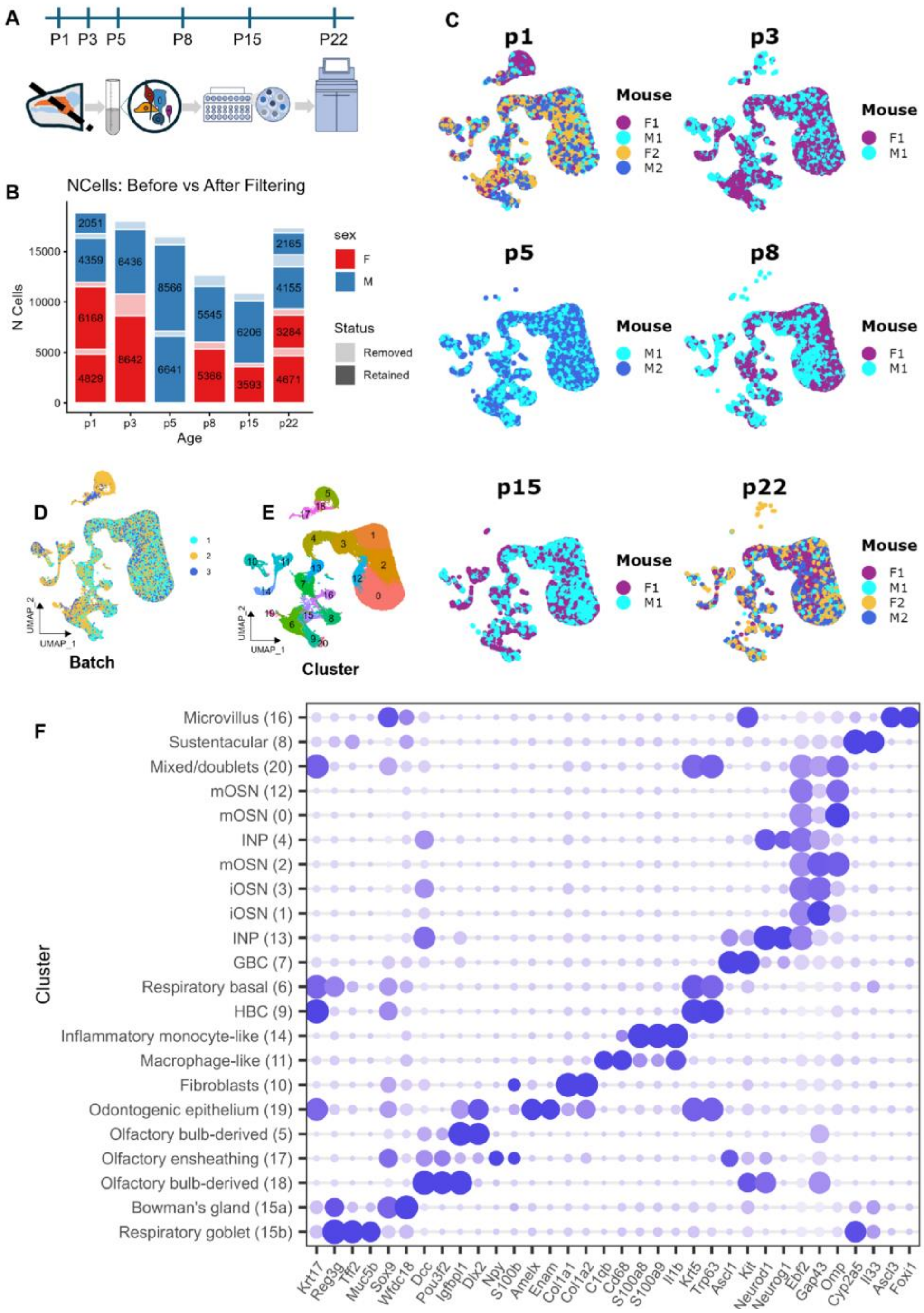

Figure S2: Single-cell RNA-seq processing, quality control, and clustering overview. (A) Overview of single cell processing pipeline. **(B)** Metadata indicating ages and sexes of removed cells after QC filtering. **(C)** UMAPs colored by animal, split by age show good inter-sample mixing, with no clear separation by animal. **(D)** UMAP colored by dissection batch. Batch 2 (yellow), containing P1 animals, has relatively more respiratory epithelium and olfactory bulb than other batches due to the difficulty of dissecting these small animals. **(E)** UMAP colored by cluster. **(F)** Dotplot of marker genes by cluster.

bioRxiv preprint doi: https://doi.org/10.64898/2026.07.28.741343; this version posted July 29, 2026. The copyright holder for this preprint (which was not certified by peer review) is the author/funder. This article is a US Government work. It is not subject to copyright under 17 USC 105 and is also made available for use under a CC0 license.

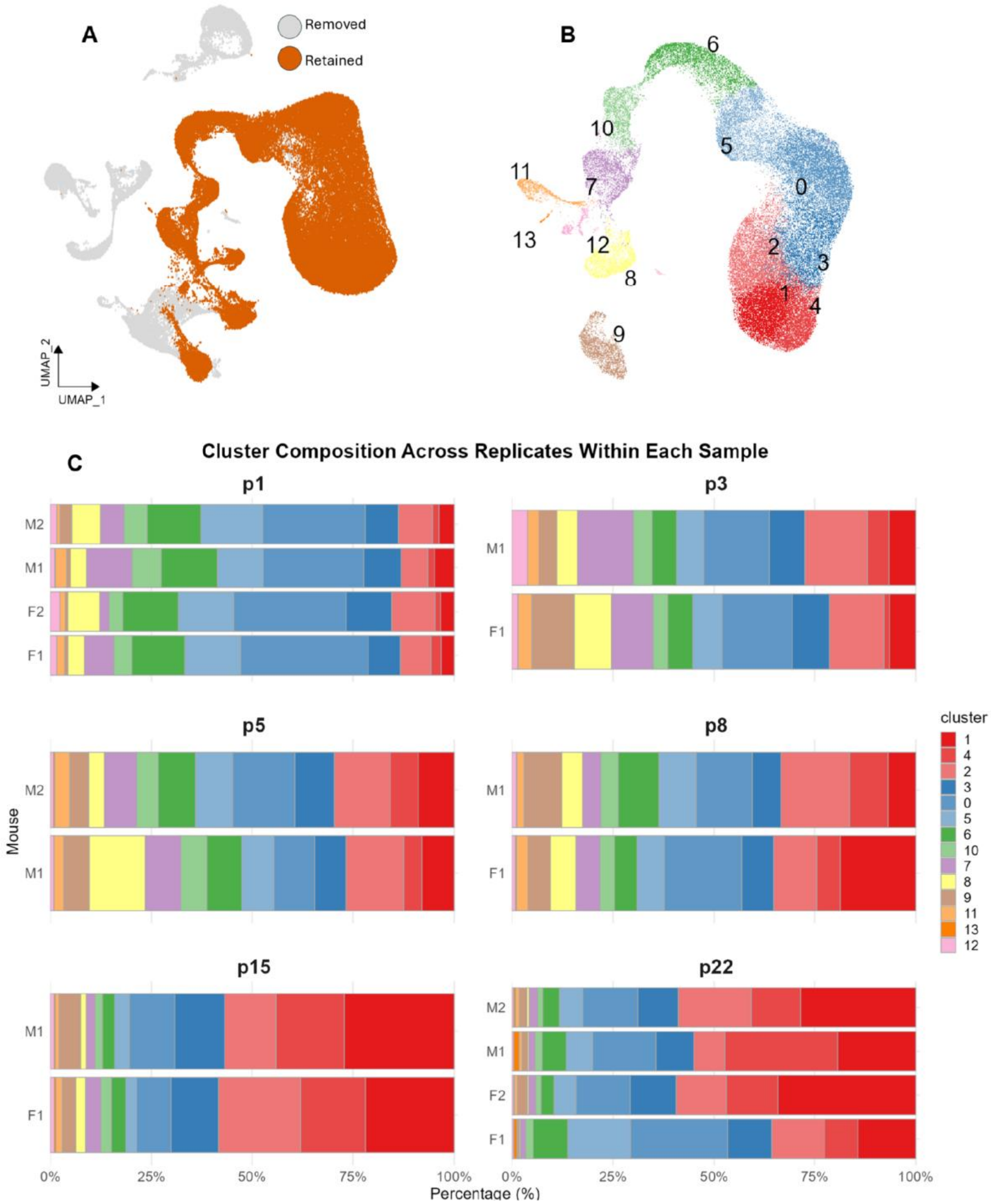

Figure S3: Sensory OE cell types by sample. (A) UMAP of whole OE showing cells removed (grey, non-sensory) and retained (orange, sensory) for downstream processing. **(B)** UMAP of sensory OE labeled by cluster. **(C)** Stacked bar plots showing the percentage of cells from each animal in each cluster.

bioRxiv preprint doi: https://doi.org/10.64898/2026.07.28.741343; this version posted July 29, 2026. The copyright holder for this preprint (which was not certified by peer review) is the author/funder. This article is a US Government work. It is not subject to copyright under 17 USC 105 and is also made available for use under a CC0 license.

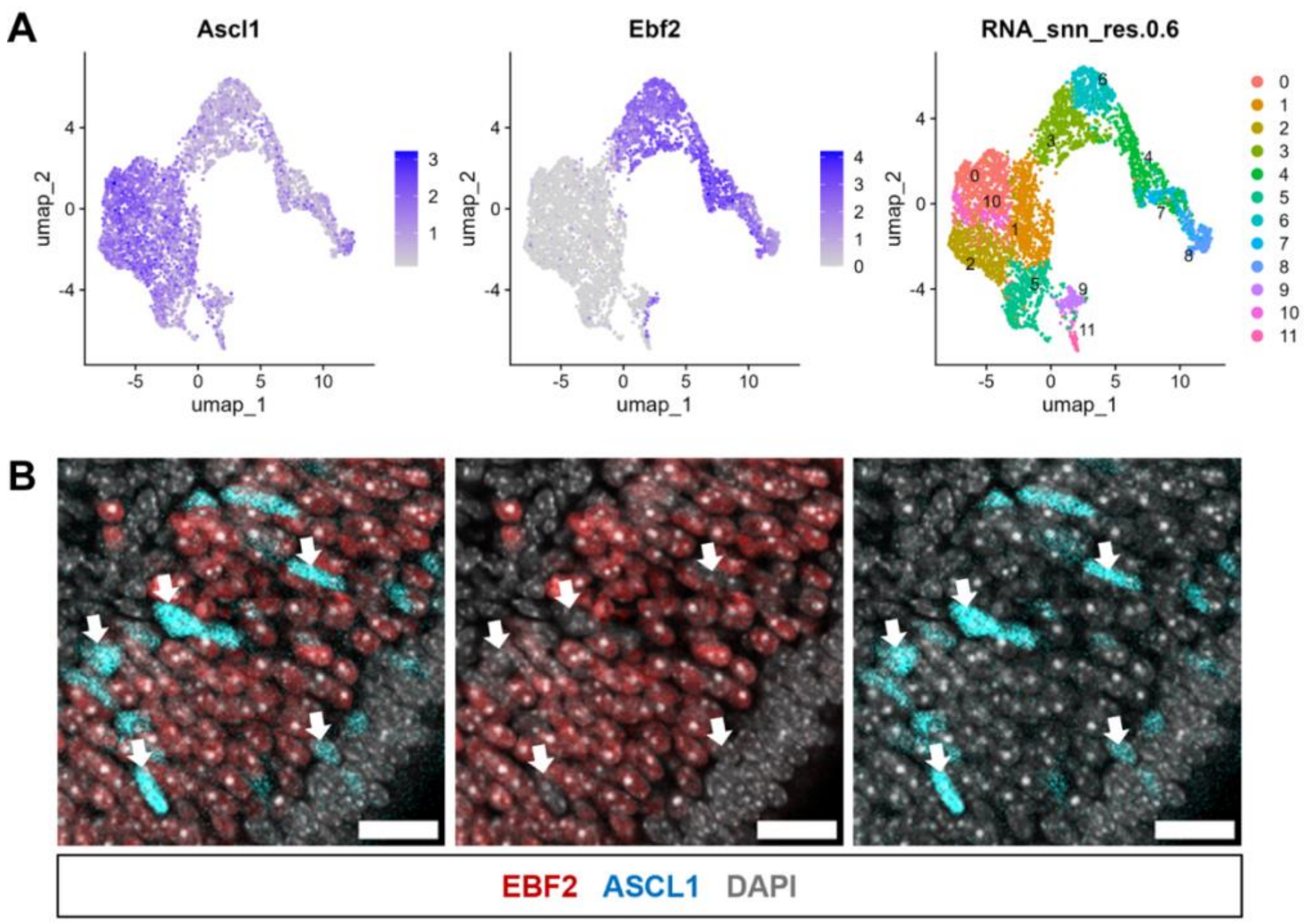

Figure S4: ASCL1 and EBF2 protein are not co-expressed at P1. (**A)** UMAPs of *Ascl1*$^{+}$ cell subset colored by *Ascl1* expression (left), *Ebf2* expression (middle), or cluster membership (right). **(B)** Representative immunofluorescence images of wild-type C57BL/6 OE at P1 showing EBF2 (red), ASCL1 (cyan), and DAPI (gray); Arrows indicate cells expressing ASCL1 but not EBF2. Scale bar: 20 μm

bioRxiv preprint doi: https://doi.org/10.64898/2026.07.28.741343; this version posted July 29, 2026. The copyright holder for this preprint (which was not certified by peer review) is the author/funder. This article is a US Government work. It is not subject to copyright under 17 USC 105 and is also made available for use under a CC0 license.

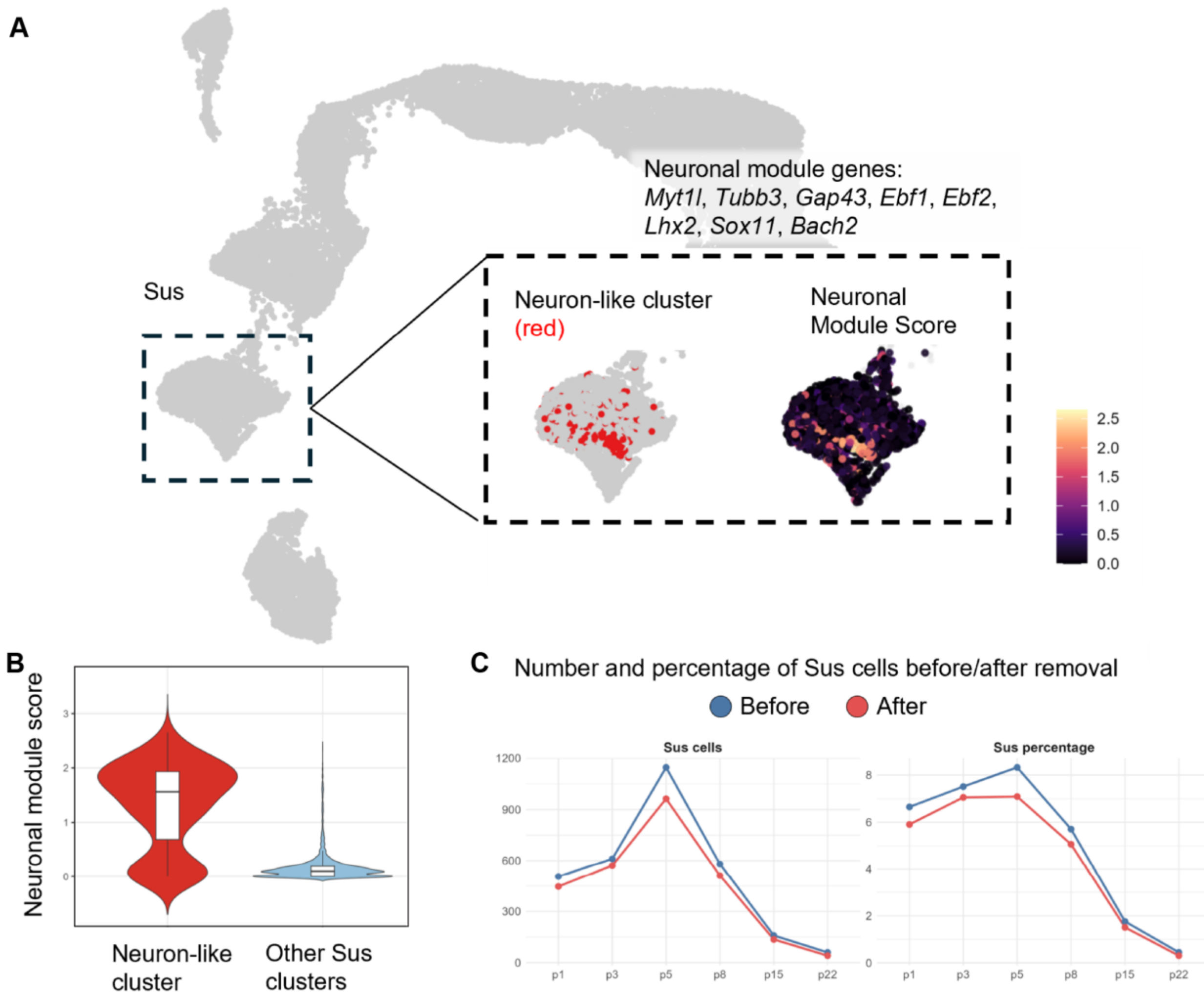

Figure S5: Identification and removal of a neuronal-gene expressing sustentacular cell cluster. (A) UMAP of sensory OE cells highlighting the sustentacular (Sus) compartment (dashed box). **(B)** Sus cells with cluster 57 highlighted in red (left) and the corresponding neuronal module score overlaid (right), showing enrichment of neuronal gene expression within cluster 57. The neuronal module was calculated from the genes *Myt1l*, *Tubb3*, *Gap43*, *Ebf1*, *Ebf2*, *Lhx2*, *Sox11*, and *Bach2*. **(C)** Violin plot comparing neuronal module scores in cluster 57 versus all other Sus clusters. **(D)** Number and percentage of Sus cells before and after removal of cluster 57 across developmental time points.

bioRxiv preprint doi: https://doi.org/10.64898/2026.07.28.741343; this version posted July 29, 2026. The copyright holder for this preprint (which was not certified by peer review) is the author/funder. This article is a US Government work. It is not subject to copyright under 17 USC 105 and is also made available for use under a CC0 license.

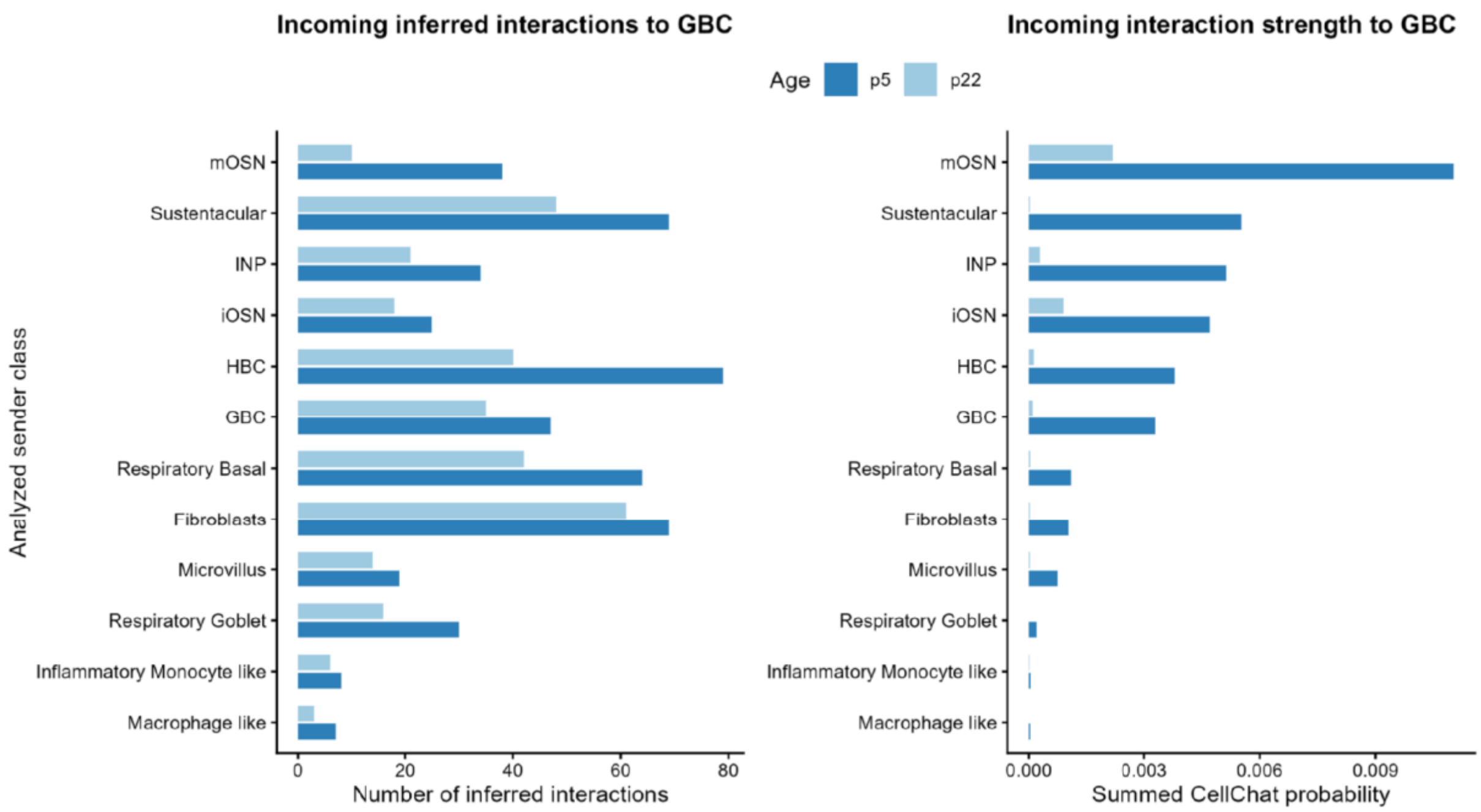

Figure S6: Summary of incoming signals to GBCs from all sender cell types considered. (Left) Number of inferred incoming interactions to GBC from senders; (Right) summed CellChat interaction strength from each sender class. Predicted incoming signaling was strongest from mOSNs, Sus cells, INPs, iOSNs, HBCs, and GBCs, whereas respiratory, immune, MV, and fibroblast inputs contributed comparatively weak signals.
